# Supplementary material for: LINC complex-Lis1 interplay controls MT1-MMP matrix digest-on-demand response for confined tumor cell migration
Source: Nat Commun. 2018 Jun 22;9:2443. doi: 10.1038/s41467-018-04865-7 (PMC6015082; doi:10.1038/s41467-018-04865-7)
Supplement: Supplementary file 1 — Supplementary Information [file 41467_2018_4865_MOESM1_ESM.pdf]

SUPPLEMENTARY MATERIAL

LINC complex-Lis1 interplay controls MT1-MMP matrix digest-on-demand response for confined tumor cell migration

Elvira Infante, Alessia Castagnino, Robin Ferrari, Pedro Monteiro, Sonia Agüera-González, Perrine Paul-Gilloteaux, Mélanie J. Domingues, Paolo Maiuri, Matthew Raab, Catherine M. Shanahan, Alexandre Baffet, Matthieu Piel, Edgar R Gomes and Philippe Chavrier

The Supplementary Material includes 5 Supplementary Figures, 1 Supplementary Table and 6 Supplementary Movies

Supplementary Figure 1 accompanying Figure 1

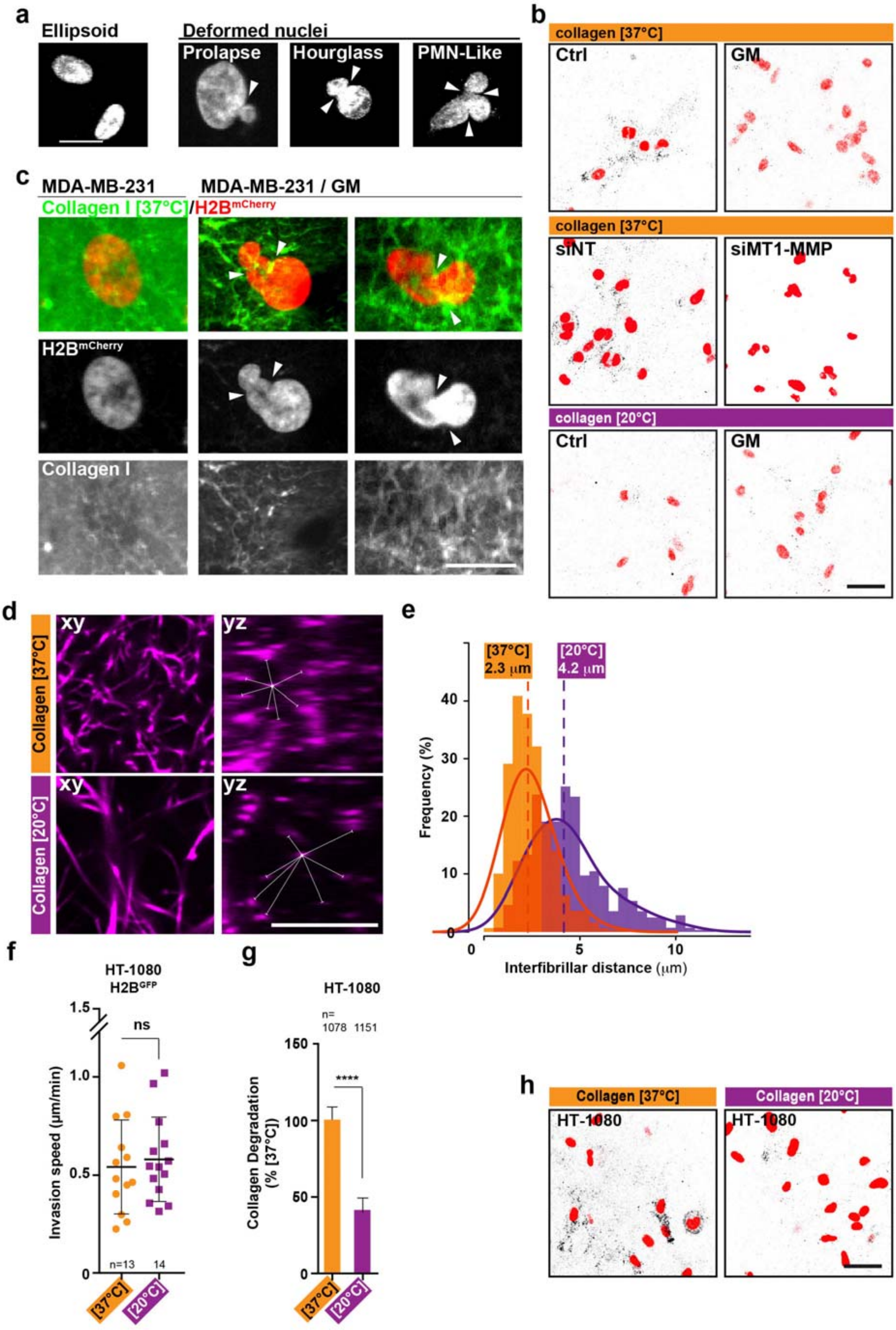

Infante et al. Supplementary Figure 1

(a) Representative examples of DAPI-stained nuclei during confined migration of MDA-MB-231 cells in 3D 37°C-polymerized collagen gel showing scored nuclear deformations (see Fig. 1d, j and Fig. 3c). Scale bar, 10 μm. **(b)** Representative images of pericellular collagenolysis detected with Col1-$^{3/4}$C antibody (black signal in the inverted images). Nuclei were stained with DAPI (red). Scale bars, 50 μm. **(c)** MDA-MB-231 cells expressing $H2B^{mCherry}$ were embedded in 3D fluorescently-labeled 37°C polymerized gel and treated with GM6001 (GM) or not and analyzed by confocal spinning disk microscopy. Arrows indicate confining collagen fibrils. Scale bar, 10 μm. **(d)** Confocal xy and yz (through dashed line in xy planes) sections of fluorescently-labeled 2.0 mg/ml type I collagen gels polymerized at 37°C or 20°C. Scale bar, 10 μm. **(e)** Inter-fibril distance distribution estimated from xy, xz and yz optical planes in 3D large and small pore size collagen gels. **(f)** Invasion speed of HT-1080 cells expressing $H2B^{GFP}$ as in Fig. 1e. Unpaired t-test. **(g, h)** Pericellular collagenolysis by HT-1080 cells as in Fig. 1f and representative images (panel h, scale bar, 50 μm). *n,* number of cells analyzed from three independent experiments; Mann-Whitney test.

Supplementary Figure 2. Effect of ^{GFP}LMNA overexpression in MDA-MB-231 cells

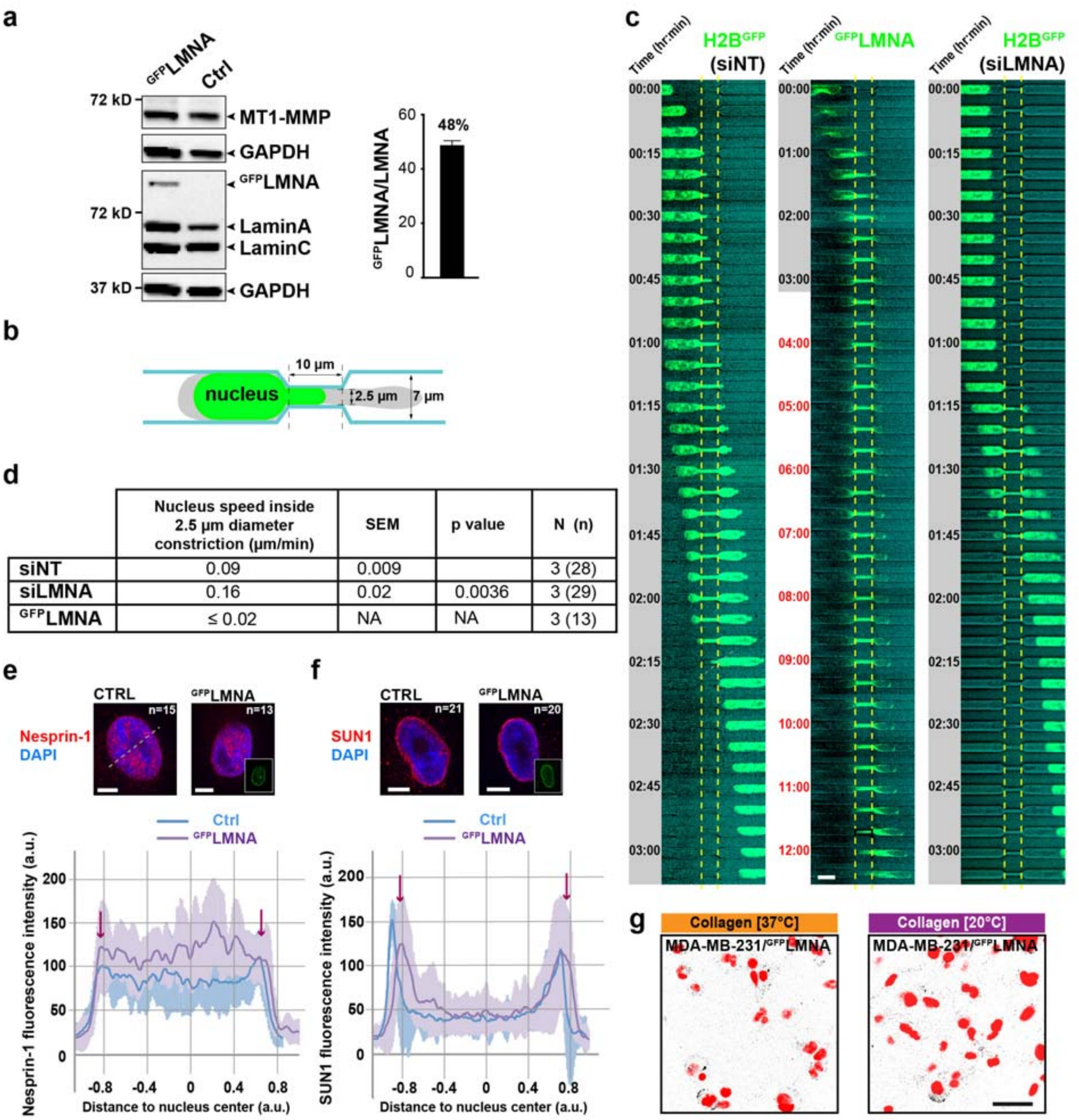

(a) Representative immunoblots for MT1-MMP and laminA, laminC and ^{GFP}LMNA expression level with GAPDH as loading control. Cell lysates were prepared from control or ^{GFP}LMNA-overexpressing MDA-MB-231 cells. The graph is the quantification of ^{GFP}LMNA expression in comparison to endogenous LMNA level from three independent experiments. **(b)** Schematic overview of the microfabricated PDMS channels with 2.5 μm-diameter constriction. **(c)** Image galleries from

representative time-lapse sequence of ^{GFP}H2B-overexpressing MDA-MB-231 cells treated with control (siNT) or LMNA siRNA (siLMNA) or overexpressing ^{GFP}LMNA migrating inside a 7-μm diameter microchannel including 2.5-μm diameter constrictions (delimited by yellow dashed lines). Time is in hr:min (note different time scale for ^{GFP}LMNA-positive cell). Scale bar, 10 μm. **(d)** Mean nucleus speed within 2.5 μm-diameter constriction ± SEM (μm/min); N, number of independent experiments; *n,* number of tracked nuclei. Mann-Whitney test. NA, not applicable (out of thirteen ^{GFP}LMNA-positive nuclei analyzed, two crossed through the 2.5 μm-diameter constriction with a speed of 0.02 μm/min and eleven nuclei stalled during the 12-h movie). **(e, f)** Averaged nesprin-1 (red signal in representative images in panel e) or SUN1 intensity profiles (red signal in representative images in panel f) ± SD from MDA-MB-231 cells expressing ^{GFP}LMNA or not along a line-scan across the nucleus (white dashed line). Arrows in the intensity profiles point to nuclear rim. Insets in the images show GFP signals. *n*, number of cells analyzed from two independent experiments. Scale bars, 5 μm. **(g)** Representative images of pericellular collagenolysis by ^{GFP}LMNA-expressing MDA-MB-231 cells in small and large pore size collagen gels detected with Col1-$^{3/4}$C antibody (black signal in the inverted images). Nuclei were stained with DAPI (red). Scale bars, 50 μm.

Supplementary Figure 3 accompanying Figure 2

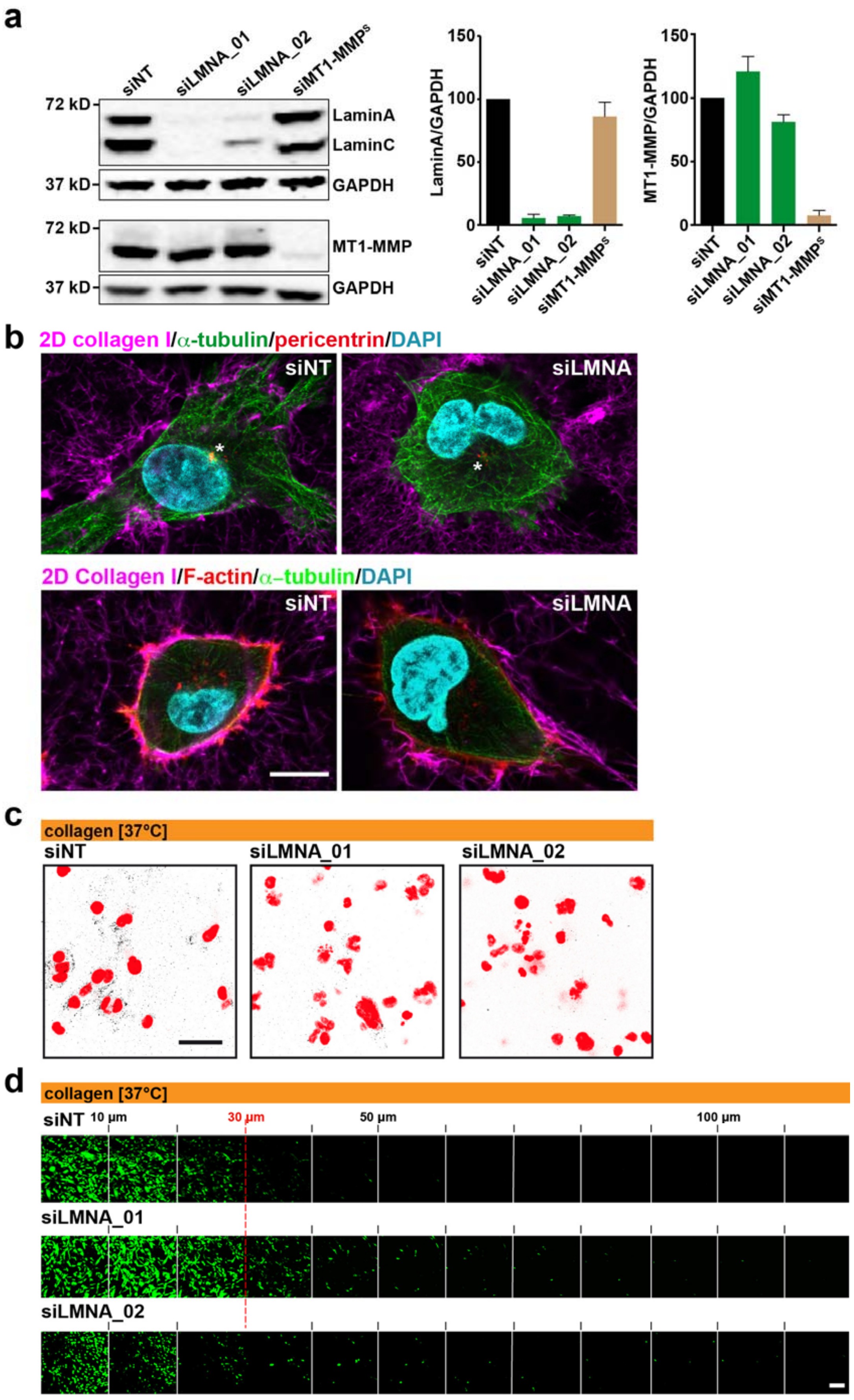

(a) Representative immunoblots for LaminA/LaminC (upper row) and MT1-MMP (lower row) with GAPDH as loading control. Cell lysates were prepared from MDA-MB-231 cells silenced for LMNA or MT1-MMP by siRNA treatment as indicated. Graphs represent LaminA and MT1-MMP expression relative to GAPDH in siRNA-treated cells from three independent experiments. **(b)** MDA-MB-231 cells treated with control (siNT) or LMNA siRNA were plated on top of a thick layer of fluorescently-labeled type I collagen fibrils (magenta), fixed after 90 min and stained for α-tubulin (green) and centrosomal pericentrin (red, asterisks) and nucleus (cyan, upper row), or for F-actin (red) and nucleus (cyan, lower row). Scale bar, 10 μm. **(c)** Pericellular collagenolysis detected using anti-Col1-$^{3/4}$C antibodies in control (siNT) or siLMNA-tretaed cells in small pore size collagen gels. Scale bars, 50 μm. **(d)** Representative images from invasion assays obtained from three independent experiments. Scale bar, 100 μm

Supplementary Figure 4. Phenotypic consequences of dominant inhibitory DN-KASH expression

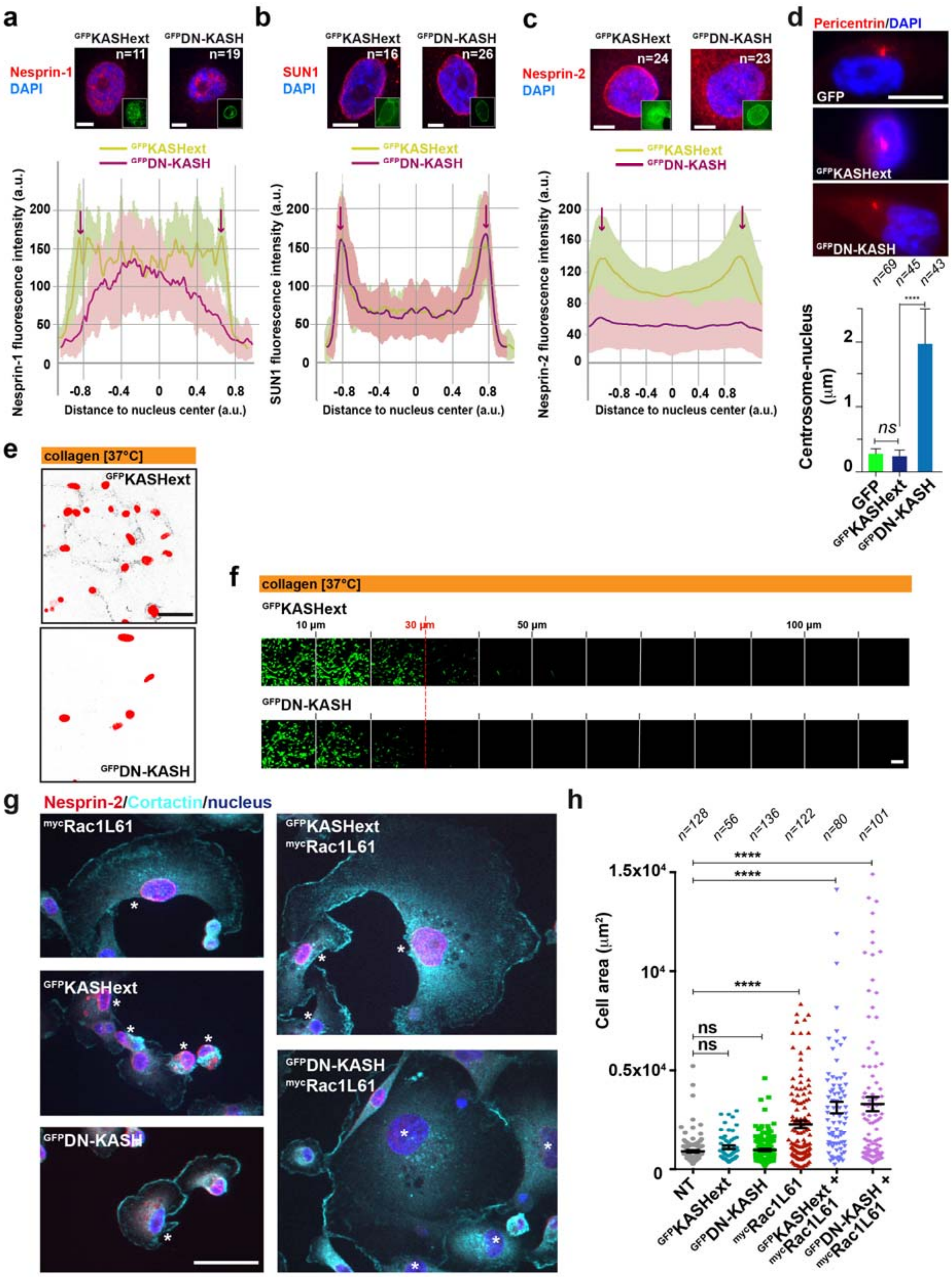

(a, c) Averaged Nesprin-1 (red signal in representative images in panel a), SUN1 (red signal in representative images in panel b) or Nesprin-2 (red signal in representative images in panel c) intensity profiles ± SD from MDA-MB-231 cells expressing ^{GFP}DN-KASH or ^{GFP}KASHext constructs along a line-scan across the nucleus. Arrows in the intensity profiles point to nuclear rim. Insets in the images show GFP signals. *n*, number of cells analyzed from two independent experiments. Scale bars, 5 μm. **(d)** Pericentrin and DAPI immunostaining (GFP signal is omitted). Scale bars, 5 μm. Mean centrosome-nucleus distance (μm) ± SEM in MDA-MB-231 cells expressing ^{GFP}DN-KASH or ^{GFP}KASHext in 3D small pore size collagen gel; *n,* number of cells analyzed from two independent experiments; Kruskal-Wallis test. **(e)** Pericellular collagenolysis (black signal in the inverted images) of ^{GFP}KASH-positive cells in small pore size collagen gel. Nuclei were stained with DAPI (red). Scale bar, 50 μm. **(f)** Representative images from invasion assays obtained from three independent experiments. Scale bar, 100 μm. **(g)** MDA-MD-231 cells expressing the indicated constructs were plated on glass substratum for 16 hrs, fixed and stained for GFP (not shown), cortactin (cyan), nesprin-2 (red) and DAPI (blue). GFP-positive cells are indicated by asterisks. Scale bars, 25 μm. **(h)** Cell area of GFP- or Myc-positive cells in μm^2. *n,* number of cells analyzed from three independent experiments. Kruskal-Wallis test.

Supplementary Figure 5 accompanying Figure 5

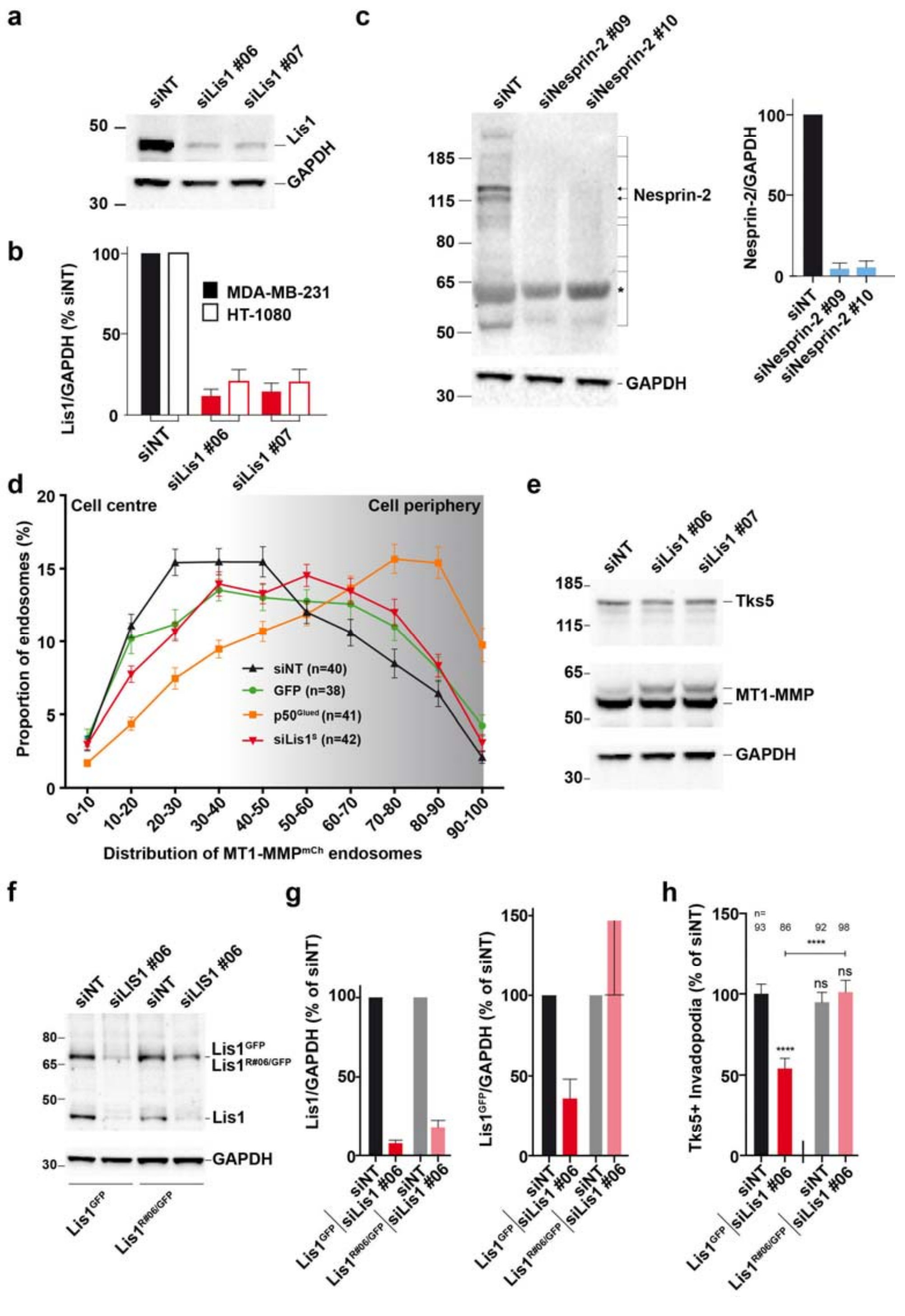

(a) Silencing of Lis1 in MDA-MB-231 cells by two independent siRNAs. **(b)** Expression of Lis1 relative to GAPDH in lysates of MDA-MB-231 and HT-1080 cells, after normalization to siNT levels from three independent experiments. **(c)** Silencing of nesprin-2 in MDA-MB-231 cells by two independent siRNAs. The graph shows

nesprin-2 expression relative to GAPDH after normalization to levels in siNT cells from three independent experiments. *, non-specific band. **(d)** Distribution of MT1-MMP^{mCh} endosomes in MDA-MB-231 cells plated on a 2D substrate. Mean percentage of MT1-MMP^{mCh}-positive endosomes according to their cell center-to-cell periphery position ± SEM; *n,* number of cells analyzed from three independent experiments. **(e)** Immunoblotting analysis showing that MT1-MMP and Tks5 expression is not affected by treatment with Lis1 siRNAs. **(f, g)** Expression of Lis1$^{GFP/R\#06}$ variant resistant to Lis1 siRNA #06. Graphs in panel g show the quantification of endogenous Lis1 (left) or overexpressed Lis1^{GFP} expression (right) relative to GAPDH normalized to levels in siNT cells from three independent experiments. **(h)** Formation of Tks5-positive invadopodia in Lis1-depleted cells is rescued by siRNA-resistant variant Lis1$^{GFP/R\#06}$. The y-axis indicates the ratio of Tks5 area to the total cell area normalized to mean value in control cells (as percentage) ± SEM. *n,* number of cells analyzed from three independent experiments. Kruskal-Wallis test.

Supplementary Table 1. Commercial antibodies used for this study

Antigen	Cie	Reference	Assay	Dilution
**SUN1**	Sigma	HPA008461	IF	1/600
**Nesprin 2**	Sigma	HPA008435	IF	1/100
**MT1-MMP**	Millipore	3328	WB	1/1000
**Lamin A/C (636)**	SantaCruz Biotechnology	sc-7292	WB	1/500
			IF	1/200
**LIS-1 (H300)**	SantaCruz Biotechnology	sc-15139	WB	1/200
			IF	1/100
**GAPDH**	SantaCruz Biotechnology	sc-25778	WB	1/10000
**Coll-3/4C**	ImmunoGlobe GmbH	0217-050	IF	1/100
**Nesprin 1**	Abcam	ab24742	IF	1/500
**Pericentrin**	Abcam	ab4448	IF	1/200
**Alpha-tubulin**	Sigma	T-9026	IF	1/600
			WB	1/10000
**Tks5**	Novus Biological	NBP1-90454	IF	1/200
			WB	1/500
**Cortactin**	Milllipore	05-180	IF	1/200
			WB	1/2000
**IgG-mouse-Cy5**	Invitrogen	A31571	IF	1/500
**IgG-mouse-Cy3**	Jackson ImmunoResearch	715-165-151	IF	1/500
**IgG-mouse-A488**	Molecular Probes	A21202	IF	1/500
**IgG-mouse-Hrp**	Jackson ImmunoResearch	115-035-062	WB	1/20000
**IgG-rabbit-A488**	Molecular Probes	A11034	IF	1/200
**IgG-rabbit-Cy3**	Jackson ImmunoResearch	711-165-152	IF	1/800
**IgG-rabbit-Hrp**	Jackson ImmunoResearch	111-035-045	WB	1/10000
**Alexa Fluor 488**	Molecular Probes	A12379	IF	1/400

**phalloidin**				
**Alexa Fluor 546 phalloidin**	Molecular Probes	A22283	IF	1/200
**GFP-Booster_ATTO 488**	ChromoTek	gba488	IF	1/100

IF, Immunofluorescence; WB, Western blot.
