# Supplementary material for: LINC complex-Lis1 interplay controls MT1-MMP matrix digest-on-demand response for confined tumor cell migration
Source: Nat Commun. 2018 Jun 22;9:2443. doi: 10.1038/s41467-018-04865-7 (PMC6015082; doi:10.1038/s41467-018-04865-7)
Supplement: Supplementary file 3 — Description of Additional Supplementary Files [file 41467_2018_4865_MOESM3_ESM.pdf]

Description of Additional Supplementary Files

Supplementary Movie 1. Polarization of MT1-MMP endosomes in front of the nucleus during 3D invasion. MDA-MB-231 cells expressing H2BGFP (green) and MT1-MMPmCh (red) were embedded in 37°C polymerized 3D fibrillar collagen-I (blue) and analyzed by confocal spinning disk fluorescence microscopy. Images were taken every 5 min. The indicated time is in h:min. Scale bar, 10 µm. Representative movie out of seven from three independent experiments.

Supplementary Movie 2. Loss of MT1-MMP endosome polarity during invasion in permissive large pore size collagen. MDA-MB-231 cells expressing H2BGFP (green) and MT1-MMPmCh (red) were embedded in fibrillar 3D large pore size collagen gel (blue) polymerized at 20°C and analyzed by confocal spinning disk fluorescence microscopy. Images were taken every 5 min. The indicated time is in h:min. Scale bar, 10 µm. Representative movie out of ten from three independent experiments.

Supplementary Movie 3. Increased nuclear stiffness upon LMNA overexpression induces MT1-MMP endosome polarization in permissive 3D collagen. MDA-MB-231 cells expressing GFPLMNA (green) and MT1-MMPmCh (red) were embedded in 3D collagen-I (blue) polymerized at 20°C and analyzed by confocal spinning disk fluorescence microscopy. Images were taken every 5 min. The indicated time is in h:min. Scale bar, 10 µm. Representative movie out of nine from three independent experiments.

Supplementary Movie 4. Invasive migration of LMNA-depleted cells in the absence of MT1-MMP endosome polarization. MDA-MB-231 cells expressing H2BGFP (green) and MT1-MMPmCh (red) were treated with siRNA specific for LMNA. Cells were embedded in 37°C polymerized 3D fibrillar collagen-I (blue) and analyzed by confocal spinning disk fluorescence microscopy. Images were taken every 5 min. The indicated time is in h:min. Scale bar, 10 µm. Representative movie out of eleven from three independent experiments.

Supplementary Movie 5. Polarity of MT1-MMP endosomes is impaired upon disruption of nesprin/SUN interaction. MDA-MB-231 cells expressing H2BGFP and GFPDN-KASH (nucleus + NE, green) and MT1-MMPmCh (red) were embedded in 37°C polymerized 3D small pore size collagen gel (blue) and analyzed by confocal spinning disk fluorescence microscopy. Images were taken every 5 min. The indicated time is in h:min. Scale bar, 10 µm. Representative movie out of seven from three independent experiments.

Supplementary Movie 6. Impaired polarity of MT1-MMP endosomes upon Lis1 silencing. MDA-MB-231 cells expressing H2BGFP (green) and MT1-MMPmCh (red) were treated with siRNA specific for Lis1. Cells were embedded in 37°C polymerized 3D small pore size collagen gel (blue) and analyzed by confocal spinning disk fluorescence microscopy. Images were taken every 5 min. The indicated time is in h:min.
